# Meta-Analysis of Correlations between Altmetric Attention Score and Citations in Health Sciences

**DOI:** 10.1155/2021/6680764

**Published:** 2021-04-07

**Authors:** Jafar Kolahi, Saber Khazaei, Pedram Iranmanesh, Jeehyoung Kim, Heejung Bang, Abbasali Khademi

**Affiliations:** ^1^Independent Research Scientist, Founder of Dental Hypotheses, Isfahan, Iran; ^2^Department of Endodontics, School of Dentistry, Kermanshah University of Medical Sciences, Kermanshah, Iran; ^3^Department of Endodontics, Dental Research Center, Dental Research Institute, School of Dentistry, Isfahan University of Medical Sciences, Isfahan, Iran; ^4^Department of Orthopedic Surgery, Seoul Sacred Heart General Hospital, Seoul, Republic of Korea; ^5^Division of Biostatistics, Department of Public Health Sciences, School of Medicine, University of California, Davis, CA, USA

## Abstract

**Introduction:**

In recent years, several controversial reports of the correlation between altmetric score and citations have been published (range: -0.2 to 0.8). We conducted a meta-analysis to provide an in-depth statistical analysis of the correlation between altmetric score and number of citations in the field of health sciences.

**Methods:**

Three online databases (Web of Science, Scopus, and PubMed) were systematically searched, without language restrictions, from the earliest publication date available through February 29, 2020, using the keywords “altmetric,” “citation,” and “correlation.” Grey literature was also searched via WorldCat, Open Grey, and Google Scholar (first 100 hits only). All studies in the field of health sciences that reported on this correlation were included. Effect sizes were calculated using Fisher's *z* transformation of correlations. Subgroup analyses based on citation source and sampling methods were performed.

**Results:**

From 27 included articles, 8 articles comprise several independent studies. The total sample size was 9,943 articles comprised of 35 studies. The overall pooled effect size was 0.19 (95% confidence interval 0.13 to 0.26). Bivariate partial prediction of interaction between effect size, citation source, and sampling method showed a greater effect size with Web of Science compared with Scopus and Dimensions. Egger's regression showed a marginally nonsignificant publication bias (*p* = 0.055), and trim-and-fill analysis estimated one missing study in this meta-analysis.

**Conclusion:**

In health sciences, currently altmetric score has a positive but weak correlation with number of citations (pooled correlation = 0.19, 95% C.I 0.12 to 0.25). We emphasize on future examinations to assess changes of correlation pattern between altmetric score and citations over time.

## 1. Introduction

The increasing demand for investigators to disseminate their research findings on the internet has given birth to new terms such as “Twitter science stars” and “Kardashian Index” (which measures over/under Twittersphere activity) [1, 2]. A profusion of web-based technologies facilitates quantification of research impact. Alternative metrics, or “altmetrics,” are described as new and emerging concepts that can complement traditional citation-based bibliometrics [3–5]. Sources of altmetric data include the following: social media such as Twitter and Facebook (mentions on public pages only), Google+ and Reddit, news outlets, scientific blogs, policy documents, patents, Wikipedia, video uploaders such as YouTube, sites running Stack Exchange (Q&A), Publons and Faculty of 1000 Prime, and reference managers such as Mendeley [6]. Currently, there are several altmetric data providers including the *Altmetric Institution* (http://altmetric.com/), *Plum Analytics* (http://plumanalytics.com/), and *ImpactStory* (http://impactstory.org/). Several academic publishers, including John Wiley & Sons, Inc., Journal of the American Medical Association (JAMA) Network, Taylor & Francis, Springer Nature, and Elsevier Publishing use one of these resources.

The *Altmetric Institution* is the most widely used provider. Altmetric database traced more than 27 million research output (article, book, book chapter, clinical trial record, and data set) and found more than 14 million research output with online attention and 122 million total altmetric mentions. As defined by the Altmetric Institution “*altmetric score is a weighted count of all of the mentions Altmetric has tracked for an individual research output, and is designed as an indicator of the amount and reach of the attention an item has received.*” [7]. It does not employ equal weighting values for the various altmetric data resources when estimating the altmetric score (weighting algorithm available at http://bit.ly/3ra2ImZ). For instance, when analyzing the article, “Characteristics of and Important Lessons from the Coronavirus Disease 2019 (COVID-19) Outbreak in China” [8] (*JAMA*, Feb 2020) using altmetrics, it stands at the top 5% of all research outputs (altmetric score: 4039). It was discussed in 47 mainstream news outlets, 8 scientific blogs, 5745 tweets (with an upper bound of 14,113,932 followers), 20 Facebook pages, 1 Wikipedia article, and 5 Reddit posts (http://www.altmetric.com/details/76606927).

Research funders and charitable organizations such as the Wellcome Trust and John Templeton Foundation are paying attention to altmetric analysis [9]. Steve Fitzmier, Templeton Foundation's Director of the Planning and Evaluation, stated, *“At the core of the Foundation's mission is a desire to both fund high quality research and to generate greater public engagement with the research we support;... While analyzing metrics such as citations can be helpful to assess impact, these methods provide an incomplete picture.”* [10]. A good example in support of the previous statement is a study on the influence of the alcohol industry on alcohol policy [11]. The Wellcome Trust invested in the study, which alleged that several submissions to the Scottish Government misrepresented research outputs so as to support policies preferential to the alcohol industry. Three months following its publication in PLOS Medicine, it remained without citation. Yet, altmetrics revealed that the article had been tweeted by key influencers, including members of the European Parliament, international nongovernmental organizations, and a sector manager for Health, Nutrition, and Population at the World Bank, demonstrating its impact in the policy sphere worldwide [9].

**Figure 1:**
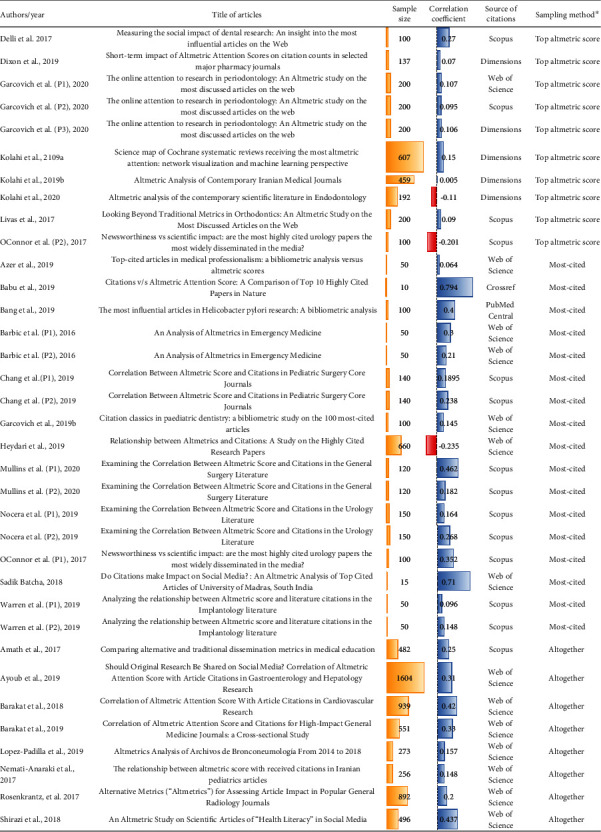
Characteristics of 35 included studies. ^∗^Correlation between altmetric score and number of citations examined among (1) articles with the highest altmetric score (top altmetric score), (2) articles with the highest citation rate (most-cited), and (3) altogether articles in specific field and year (altogether). P stands for part.

Google trend analysis showed that, over the past five years, the search term “altmetric” received more attention than “bibliometric” worldwide [12]. Traditional citation-based bibliometrics accrue slowly. It was reported that only 50% of articles are cited in the first three years following publication or 26 years after publication for some [13]. In contrast, common altmetric data resources are updated in real time (e.g., Twitter and Wikipedia) or on a daily basis (e.g., Facebook and Google+). However, the relationship between altmetrics and citations is a challenging and controversial issue among researchers. Recently, several controversial results have been published (Figure 1) regarding this correlation; thus, the aim of the present meta-analysis was to estimate the correlation between altmetrics and citations in the field of health sciences.

## 2. Methods

### 2.1. Formatting the Review Question and Outcome

The pooled correlation coefficient between altmetric score and citation numbers in health sciences research was the primary outcome of interest in the present meta-analysis.

### 2.2. Search Strategy

Three online databases (Web of Science, Scopus, and PubMed) were systematically searched, without language restrictions, from the earliest publication date available through February 29, 2020, using the keywords “altmetric^∗^”, “citation^∗^”, and “correlation,” where the asterisk “∗” was used as a truncation symbol. Grey literature was also searched via WorldCat, Open Grey, and Google Scholar (first 100 hits only). Additionally, references of all included studies were also searched manually.

### 2.3. Study Eligibility Criteria

All studies in the field of health sciences that reported the correlation between altmetric score (Altmetric LLP, London, UK) and number of citations were included in this meta-analysis. Web of Science, Scopus, Dimensions, Crossref, and PubMed Central were considered as reliable sources for citation number. Studies in which the exact correlation coefficient or sample size was not reported, the source for number of citations was unclear, the source of altmetrics was other than Altmetric Institute (such as PlumX Metrics), or those representing fields other than health sciences were excluded.

### 2.4. Data Collection

After identification of potentially eligible articles, duplicates were removed. Then, articles were reviewed independently by two authors (J.K. and P.I.). SWIFT-Review software (Sciome LLC, NC, USA) was also used for text mining at the screening phase. This software uses machine learning algorithms for topic modeling where abstracts relating to similar topics are automatically grouped [14]. Data (author, year, article title, sample size, correlation coefficient, source of citations, and sampling method) were extracted independently by two authors (J.K. and P.I.) and recorded on a standard data collection sheet. Disagreements were resolved via the Delphi technique at each stage [15].

### 2.5. Quantitative Data Synthesis

Effect sizes were calculated using Fisher's *z* transformation of correlations. The formula to transform *r* to a *z*-score is *z*′ = 0.5[ln(1 + *r*)–ln(1 − *r*)]. If statistical heterogeneity existed (*I*^2^ > 50%, *p* < 0.05) [16], data were analyzed using the random-effects model (method: restricted maximum-likelihood estimator); otherwise, the data were pooled by the fixed-effects model (method: inverse-variance). Meta-analysis was carried out by STATA 16 (StataCorp, College Station, TX), metafor (http://www.metafor-project.org/doku.php), and metacor (http://www.rdocumentation.org/packages/meta/versions/4.9-9/topics/metacor) R packages (R Foundation for Statistical Computing, Vienna, Austria). Interaction between moderators and effect size was examined by the random forest model, a machine learning algorithm, using metaforest (https://cran.r-project.org/web/packages/metaforest/index.html) R package. The random forest model is a method of regression that creates a set of decision trees consisting of a great number of separate trees, which operate as a group, like a forest. The subgroup analysis was based on citation source as well as sampling method of studies. Baujat plot was used as a diagnostic method to identify sources of heterogeneity and influential studies on the overall results [17]. The regression-based Egger's test and the Begg's rank test were employed to quantify publication bias. Nonparametric trim-and-fill analysis was used to estimate the number of studies missing from the meta-analysis.

## 3. Results

### 3.1. Characteristics of the Included Studies

**Figure 2:**
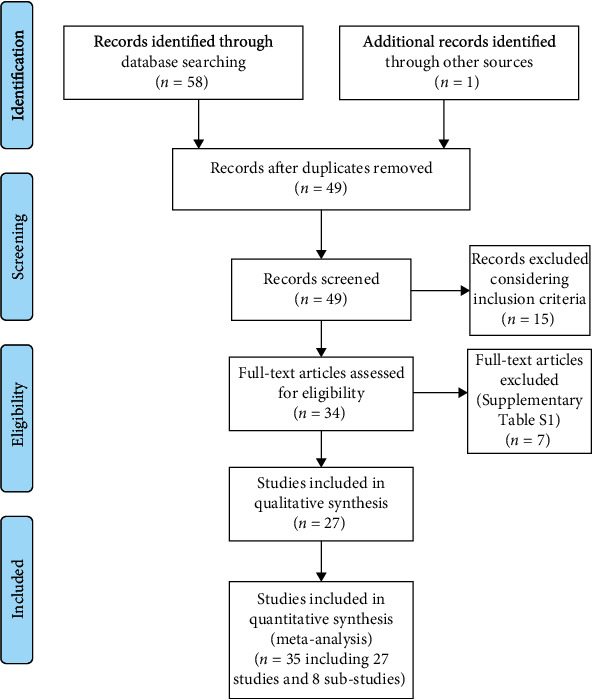
PRISMA flow diagram.

A total of 35 studies was included in the analyses (Figure 2). From 27 included articles [18–44], eight articles comprise several independent studies. For example, Barbic et al. [21] used two independent studies. They analyzed the most frequently cited emergency medicine articles published in (1) the top 10 emergency medicine journals and (2) the rest of the medical literature and reported correlation coefficient and sample size for each group independently. In addition, some studies included several substudies based on the source of citations. For instance, Garcovich et al. [30] reported three correlation coefficients, one each for Web of Science, Dimensions, and Scopus. Finally, characteristics of 35 included studies and seven excluded studies are provided in Figure 1 and Supplementary Table S1.

**Figure 3:**
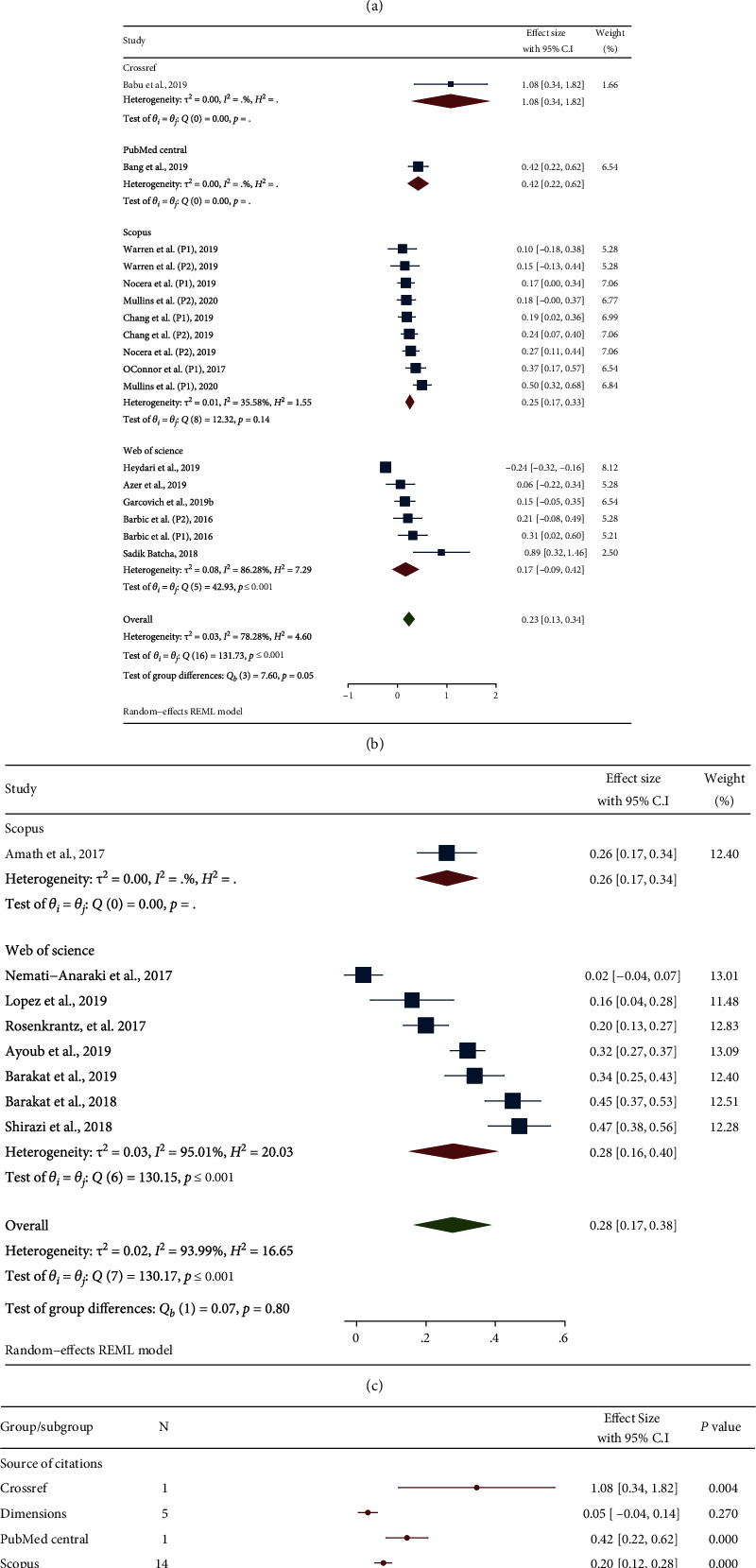
Forest plots showed results of meta-analysis of correlation between the altmetric score and citations among articles: with the highest altmetric score (a), with the highest citations rate (b), altogether articles in specific field and year (c) and all included studies in meta-analysis (d). Readers must note when Fisher's z transformation used for effect size calculation if the correlation was less than or equal to 0.3, the effect size would be asymptotically equal to correlation (if r ≤ 0.3 then r ≃ effect size). For more details, please see https://www.statisticshowto.com/fisher-z/ (REML: restricted maximum-likelihood).

The total sample size was 9,943 articles from various health disciplines. Studies used different databases as the source of number of citations, most commonly Web of Science and Scopus (Figure 3). Furthermore, three different sampling methods were recognized among the included studies. Correlation between altmetric score and number of citations was examined among the following: (1) articles with the highest altmetric score (*n* = 10), (2) articles with the highest number of citations (*n* = 17), and (3) altogether articles in specific field and year (*n* = 8). While almost all studies showed positive correlation coefficients, in three studies, the coefficient was negative (Figure 1).

### 3.2. Meta-Analysis Results

The pooled correlation coefficient was 0.19 (95% confidence interval (C.I) 0.12 to 0.25), and the pooled effect size was 0.19 (95% C.I 0.13 to 0.26). Subgroup analyses based on citation source as well as sampling method of study are reported in Figure 3. By dividing the groups based on sampling method, the highest degree correlation was found among studies in which their sample involved altogether articles in specific field and year (pooled effect size = 0.28, 95% C.I 0.17 to 0.38), followed by articles with the highest number of citations and articles with the highest altmetric score. Considering the source of databases for counting number of citations (except PubMed Central and Crossref, which each involved only one study), the highest pooled correlation coefficient was found for the Web of Science group (pooled effect size = 0.22, 95% C.I 0.10 to 0.33), followed by Scopus and Dimensions (Figure 3).

**Figure 4:**
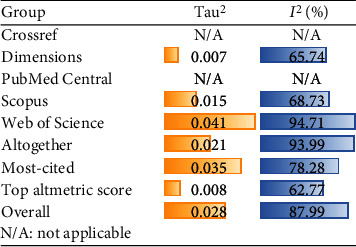
Heterogeneity assessment summary. Readers must note PubMed Central and Crossref groups involve only one study.

General heterogeneity was high for all included studies (*I*^2^ = 87.99%; Figure 4). The Baujat plot showed that Heydari et al. [24] had the greatest impact on overall meta-analysis output and overall heterogeneity [45].

**Figure 5:**
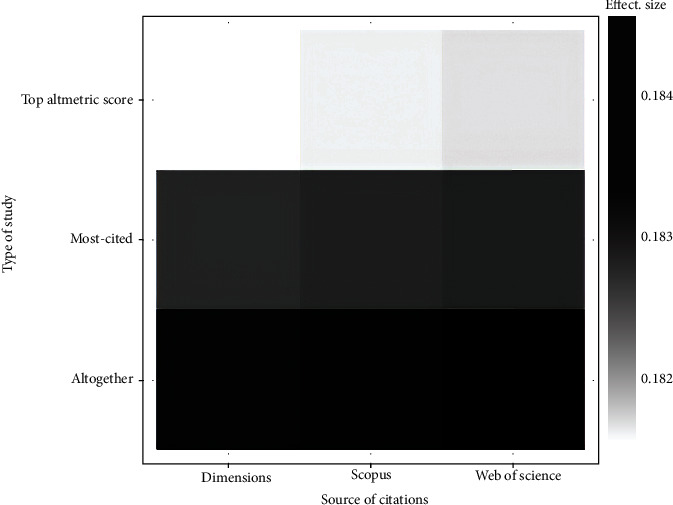
Binned scatter plot of the bivariate partial prediction of interaction between effect size, source of citation, and type of study. This plot created by means of random forests model (a machine learning algorithm) (Number of trees in forest: 500, Minimum terminal node size: 5). Readers must note Crossref and PubMed Central groups, each one involved only one article. So for better clarification, they were removed from the analysis. Please see Figure 1 for more details about sample sizes. Analysis was carried out via https://cjvanlissa.shinyapps.io/MetaForest_online/.

Bivariate partial prediction of interaction between effect size, source of citation, and sampling method [46] showed that effect size was greater when the citation source was Web of Science, compared to Scopus and Dimensions (Figure 5).

**Figure 6:**
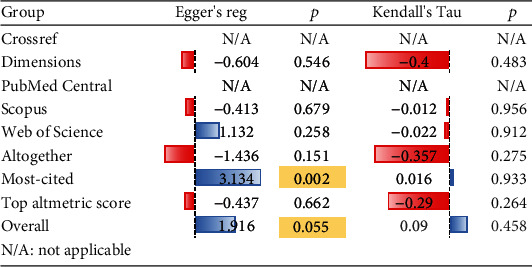
Publication bias assessment using the regression-based Egger's test and the Begg's rank test. Readers must note PubMed Central and Crossref groups involve only one study.

**Figure 7:**
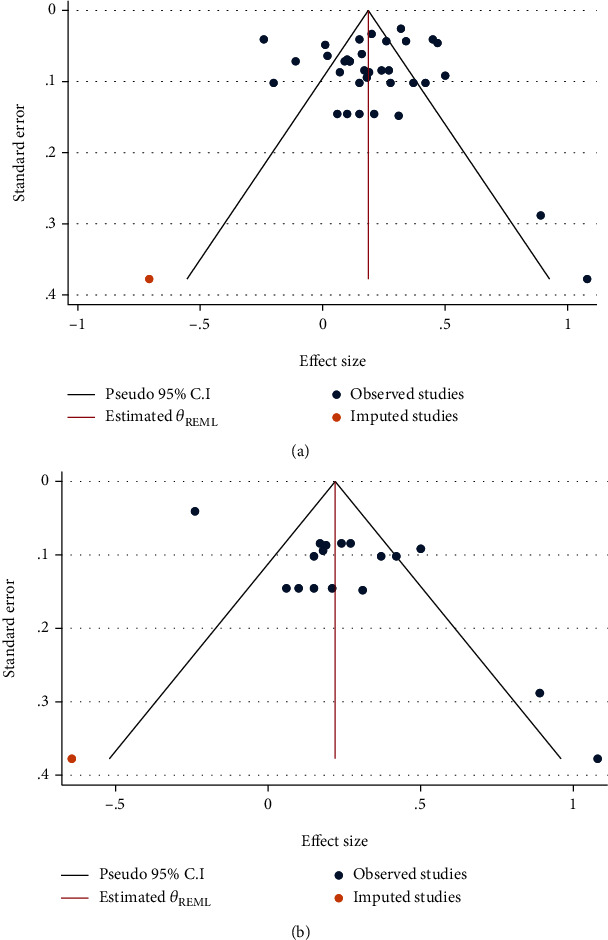
Funnel plots with nonparametric trim-and-fill analysis of publication bias for two groups which involved publication bias comprising all included study groups (a) and the most cited group (b). The linear estimator was used to estimate the number of missing studies. The observed effect size for all included studies was 0.192 (95% confidence interval 0.128 to 0.255), and the observed + imputed effect size was 0.186 (95% confidence interval 0.122 to 0.250) (a). The observed effect size for the most cited group was 0.233 (95% confidence interval 0.127 to 0.340), and the observed + imputed effect size was 0.220 (95% confidence interval 0.112 to 0.327) (b). Please see Figure 6 for more details about publication bias assessments (REML: restricted maximum-likelihood).

Egger's regression showed publication bias among the most-cited group (*p* = 0.002) and a marginally nonsignificant result for all included studies in meta-analyses (*p* = 0.055) (Figure 6). Nonparametric trim-and-fill analysis showed that there could be potentially one missing study among all included studies in meta-analysis and the most-cited group (Figure 7).

## 4. Discussion

It is estimated that there are more than 3.8 billion active social media users globally. The biomedical research community and professional healthcare providers increasingly use social media to disseminate information to other researchers, practitioners, and members of the public. Well-known healthcare organizations and high-impact biomedical journals are active in the Twittersphere; for example, @MayoClinic has 1.9M followers and 50.3K tweets, and @TheLancet has 463.6K followers and 15.1K tweets. A PubMed query on June 24, 2020, with search term “altmetric” [All Fields] showed a rapidly increasing number of publications after 2014 (number of articles = 7.7333∗year–15560, *R*^2^ = 0.84). Yet the question, “*Is altmetric score correlated with citations?*” remains.

To our knowledge, this is the first in-depth meta-analysis to elucidate the correlation between altmetric score and number of citations. This study showed a weak, positive, linear correlation (pooled correlation = 0.19). Yet, it should be noted that altmetrics is a relatively new concept among scholars and future rise in awareness and use of social media among researchers may increase this correlation.

This meta-analysis involved a high level of heterogeneity (*I*^2^ = 87.99%), which we tried to assess by subgroup analyses based on the source of citation number and sampling method (Figure 1). Included studies were from different fields of health sciences research including internal medicine, plastic surgery, urology, radiology, pharmacy, and dentistry, etc. In the general and internal medicine fields, very high impact and influential journals exist (e.g., The Lancet, The Journal of the American Medical Association, and The New England Journal of Medicine). These journals have a strong presence in the Twittersphere, and articles published therein are widely covered by news outlets and social media. For example, referring to the December 2017 article published in The Lancet, “Worldwide Trends in Body-Mass Index, Underweight, Overweight, and Obesity from 1975 to 2016: A Pooled Analysis of 2416 Population-Based Measurement Studies in 128·9 Million Children, Adolescents, and Adults,” this article is in the top 5% of all research outputs scored by altmetrics (altmetric score: 4239). It was discussed in 426 news stories from 323 outlets, 16 scientific blogs, 2818 tweets (with an upper bound of 13,407,536 followers), 72 Facebook pages, 1 Google+ post, 1 Reddit post, 1 Q&A thread, and 3014 Mendeley readers (https://www.altmetric.com/details/27275391). Further analysis showed that 68% of tweets were carried out by members of the public, since obesity and fitness are popular topics. This situation is not comparable with specialized narrow fields of health sciences such as endodontology. Topics discussed in specialized fields generally receive less online attention and are rarely shared and discussed in social media and news outlets. It could be expected that the medical articles with more popular keywords and topics such as cardiovascular disease or obesity may receive more online attention and higher altmetric scores. This may be an explanation for a high level of heterogeneity in this meta-analysis.

The limitations of the present meta-analysis must be noted. Readers must note that correlation does not imply causation. Consequently, a cause and effect association between altmetric score and number of citations could not be reasonably deduced just on the basis of an observed correlation. As a suggestion for future investigations, newly developed and emerging machine learning and deep learning algorithms can be used to assess the importance and relative influence of altmetric score and related data resources (such as, Twitter, Facebook, Wikipedia, and Mendeley) on citation patterns [47]. In addition, the year (even date) of data collation for both altmetric score and number of citations must be noted. It is assumed that the number of citations and altmetric score would increase over time as researchers embrace the altmetric concept. We tried to consider year of data collection as a moderator to examine its interaction with effect size. Yet, several included studies did not report the exact data collection date. In addition, our assessments showed that year of publication was not a reliable variable due to the potential gap between data collection date and publication date. For example, Chang et al. [22] collected data in 2015, yet the articles were not published until 2019.

## 5. Recommendations for Future Researchers

With respect to emerging use of social media among scientists, we recommend future examinations to assess correlation pattern changes over time. To facilitate future meta-analysis, we encourage authors to report the following items clearly: (1) time of data collection, (2) source of citations and altmetric data, (3) number of study groups and subgroups, (4) sample size for each group and subgroup, (5) exact *r* value for each group and subgroup and correlation assessment method, (6) criteria for selection of articles such as highly cited article, and (7) type of included articles such as original research.

Below is an example to assess correlation between altmetric score and number of citations among articles related to COVID-19 pandemic which was funded by the National Institutes of Health (NIH).

PubMed was searched via the query “2019-nCoV”[All Fields] OR “COVID-19”[All Fields] OR “SARS-CoV-2”[All Fields] AND “nih funded”[Filter]. The Altmetric database (Altmetric LLP, London, UK) was used to find altmetric data. Source of citations was the Dimensions database. All data were collected/retrieved on December 13, 2020. A total of 3266 articles were found with altmetric mention (82.4%) from 3961 articles. Statistical analyses showed the following:

- Moderate correlation between altmetric score and citations (Pearson's *r* = 0.48, 95% C.I 0.45 to 0.50, *p* < 0.001) among all articles (*n* = 3266)
- Moderate correlation between altmetric score and citations (Pearson's *r* = 0.38, 95% C.I 0.29 to 0.47, *p* < 0.001) among top 10% articles with the highest altmetric score (*n* = 328)
- Moderate correlation between altmetric score and citations (Pearson's *r* = 0.41, 95% C.I 0.31 to 0.49, *p* < 0.001) among top 10% articles with the highest number of citations (*n* = 335)
- High correlation between altmetric score and citations (Pearson's *r* = 0.75, 95% C.I 0.56 to 0.86, *p* < 0.001) among systematic reviews and meta-analysis (*n* = 37)

This example indicated degree of correlation may meaningfully depend on the sampling method and characteristics of included articles (chi‐squared = 13.6, *p* = 0.004) (note: comparison between correlations was carried out via https://home.ubalt.edu/ntsbarsh/business-stat/otherapplets/MultiCorr.htm).

## 6. Conclusions

In summary, the findings of this study suggest that in health sciences research, currently altmetric score has a positive but weak correlation with number of citations (pooled correlation = 0.19, 95% C.I 0.12 to 0.25).

## Data Availability

The data of the present study are available from the corresponding author upon reasonable request.

## Supplementary Materials

Supplementary Materials Supplementary Table S1: characteristics of seven excluded studies.
