# Supplementary material for: Meta-Analysis of Correlations between Altmetric Attention Score and Citations in Health Sciences
Source: Biomed Res Int. 2021 Apr 7;2021:6680764. doi: 10.1155/2021/6680764 (PMC8046527; doi:10.1155/2021/6680764)
Supplement: Supplementary Materials — Supplementary Table S1: characteristics of seven excluded studies. [file 6680764.f1.docx]

**Supplementary Table S1:** Characteristics of seven excluded studies.


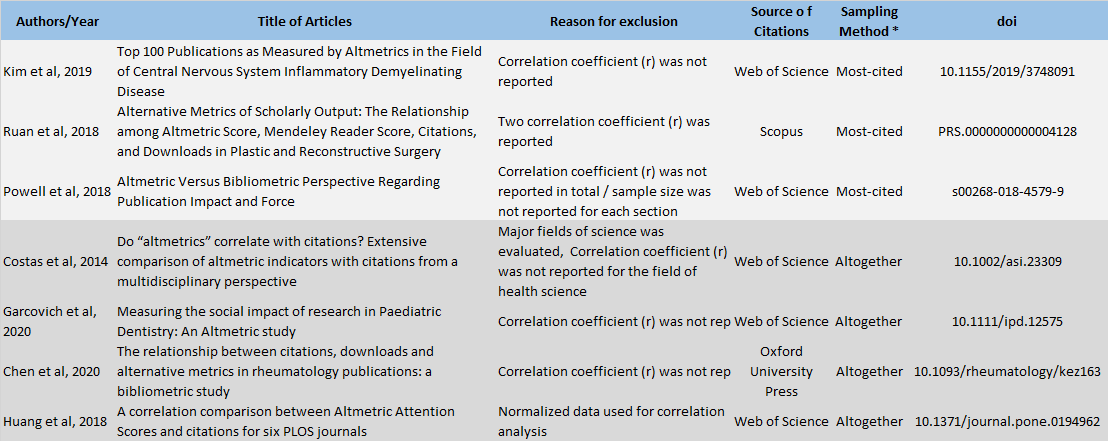


* Correlation between altmetric score and number of citations examined among 1) articles with the highest altmetric score (Top Altmetric score); 2) articles with the highest citations rate (Most-cited); and 3) altogether articles in specific field and year (Altogether).
